# Reducing Sugar, Total Phenolic Content, and Antioxidant Potential of Nepalese Plants

**DOI:** 10.1155/2020/7296859

**Published:** 2020-11-15

**Authors:** Deepa Khatri, Sumit Bahadur Baruwal Chhetri

**Affiliations:** Department of Pharmaceutical Sciences, School of Health and Allied Sciences, Pokhara University, Pokhara 33700, Nepal

## Abstract

The aim of the present study was to investigate the reducing sugar, total phenolic content, and *in vitro* antioxidant activity of 70% (*v*/*v*) ethanolic extract of seven medicinal plants grown in Nepal. The reducing sugar content and total phenolic content were determined by 3,5-dinitrosalicylic acid (DNSA) and the Folin–Ciocalteu method, respectively. The *in vitro* antioxidant activity was evaluated using the 2,2-diphenyl-1-picrylhydrazyl (DPPH) assay. The reducing sugar content of the studied plant samples ranged from 6.89 ± 2.44 to 674.13 ± 2.43 mg GE/g dry extract weight and the total phenolic content ranged from 14.87 ± 0.41 to 281.71 ± 1.47 mg GAE/g dry extract weight. The reducing sugar and total phenolic content were found highest in *Ficus glaberrima*. Antioxidant activity was found highest in *Melastoma malabathricum* (IC_50_value = 6.27 *μ*g/mL), followed by *F. glaberrima* (IC_50_value = 11.7 *μ*g/mL). A positive and significant correlation was found between (i) total phenolic content and reducing sugar content and (ii) total phenolic content and antioxidant activity. The present study is the first study for the analysis of reducing sugar content of selected plants and for the scientific exploration of *F. glaberrima*. The present result suggests that the various parts of these studied plants could be assumed as a rich source of biologically active compounds and considered beneficial for the food and pharmaceutical industries.

## 1. Introduction

The imbalance between the production of free radicals (reactive oxygen species and reactive nitrogen species) and the available antioxidants in the body leads to oxidative stress, which plays a vital role in the pathogenesis and development of various chronic and degenerative diseases such as cancer, arthritis, diabetes, aging, and cardiovascular diseases [1]. Different types of synthetic antioxidants such as butylated hydroxytoluene (BHT), butylated hydroxyanisole (BHA), propyl gallate, and tertiary butylhydroquinone (TBHQ) have been widely used in the food and pharmaceutical industries to preserve food and drug products from oxidative deterioration and used as a dietary supplement to neutralize the effect of oxidative stress inside the human body. However, this synthetic antioxidant (BHA and BHT) has been reported to induce carcinomas of forestomach in rats and hamsters [2], cytotoxicity, and apoptosis in human cells [3] and possesses adverse effects on the liver, kidney, and lung tissues [4]. Therefore, the demand for safe and potent natural plant-based antioxidants is increasing across the globe.

Antioxidant substances are abundantly present in plants in the form of phenolic compounds (e.g., phenolic acids, flavonoids, coumarins, tannins, and lignans), nitrogen compounds (e.g., alkaloids and amines), vitamins (vitamin E and vitamin C), terpenoids, and some other endogenous compounds [5–8]. These antioxidants protect cells against oxidative damage and thereby prevent the occurrence of chronic diseases such as diabetes, cancer, and cardiovascular diseases [9].

Nepal is rich in biodiversity owing to its unique geographical location and diverse climatic and altitudinal variations. The existing plant checklist of Nepal has documented around 6000 species of flowering plants and 530 ferns [10]. However, botanists have claimed that thousands of plant species are unexplored due to their extreme geographical location and availability of funding. Among the documented plant species, the medicinal potential of several plant species has not been scientifically validated. Many studies have reported the impact of diverse environmental conditions such as climate, altitude, soil type, rainfall, humidity, and degree of sun exposure on the phytoconstituents and biological activities of plant species [11–13]. Since there is tremendous geographical and climatic variation in Nepal, the chemical and biological profiles of flora and fauna found in Nepal may be varied from other places in the world. Therefore, in the present study, we selected seven Nepalese plants based on their local uses and limited scientific exploration of various parts of plants. The present study is aimed at quantitatively analyzing the reducing sugar and total phenolic content and exploring the antioxidant potential of selected plants of Nepalese origin. This research is the first to explore the reducing sugar content of selected plants and also the first scientific report on *Ficus glaberrima*.

## 2. Materials and Methods

### 2.1. Plants Collection

The fresh leaves, stems, tubers, grains, and bark of different plants were collected in July 2016 from Kaski district, Nepal. The samples were authenticated with the help of botanists and literature comparison. The voucher specimen (Table 1) was deposited at the Laboratory of Pharmacognosy, Department of Pharmaceutical Sciences, Pokhara University.

### 2.2. Extraction of Samples

The collected plant samples were cleaned and shade dried at room temperature. The dried plant samples (20 g) were grounded into coarse powder and macerated twice with 70% ethanol at a ratio of 1 : 7 *w*/*v* at 25°C for 48 h. After maceration, the solvent was evaporated under reduced pressure by using a rotary evaporator (Buchi Rotavapor, Germany).

### 2.3. Measurement of the Extraction Yield

The weight of dried extract of each plant sample was measured, and the yield percentage was calculated using the following formula:

(1) $$\begin{array}{c} \text{Extract}\text{yield}\left(\%\right)=\frac{\text{Weight}\text{of}\text{dried}\text{extract}}{\text{Weight}\text{of}\text{dried}\text{plant}\text{samples}}\times 100. \end{array}$$

### 2.4. Reducing Sugar Content (RSC)

The reducing sugar content (RSC) was determined using the 3,5-dinitrosalicylic acid (DNSA) method. The measurement was performed according to the procedure of Krivorotova and Sereikaite [22] with slight modification. DNSA reagent was prepared by dissolving 1 g of DNSA and 30 g of sodium-potassium tartaric acid in 80 mL of 0.5 N NaOH at 45°C. After dissolution, the solution was cooled down to room temperature and diluted to 100 mL with the help of distilled water. For the measurement, 2 mL of DNSA reagent was pipetted into a test tube containing 1 mL of plant extract (1 mg/mL) and kept at 95°C for 5 min. After cooling, 7 mL of distilled water was added to the solution and the absorbance of the resulting solution was measured at 540 nm using a UV-VIS spectrophotometer (Shimadzu UV-1800). The reducing sugar content was calculated from the calibration curve of standard D-glucose (200-1000 mg/L), and the results were expressed as mg D-glucose equivalent (GE) per gram dry extract weight.

### 2.5. Total Phenolic Content (TPC)

The total phenolic content (TPC) of the different plant extracts was determined using Folin–Ciocalteu reagent, following the method of Singleton and Rossi [23] with slight modification. In brief, 1 mL of crude extract (1 mg/mL) was mixed with 1 mL of Folin–Ciocalteu reagent followed by the addition of 5 mL of distilled water in a volumetric flask. After 5 min, 1 mL of sodium carbonate (10% *w*/*v*) was added and shaken vigorously. Then, the final mixture was incubated in the dark condition at room temperature for 60 min, and the absorbance was measured against the blank at 725 nm using a UV-VIS spectrophotometer. The total phenolic content of plant samples was calculated from the calibration curve of standard gallic acid (10-250 mg/L) and expressed as mg gallic acid equivalent (GAE) per gram dry extract weight.

### 2.6. Antioxidant Activity

The 2,2-diphenyl-1-picrylhydrazyl (DPPH) radical scavenging activity of plant samples was measured according to the method described by Blois [24] with slight modification. Different concentrations of plant extracts (1 *μ*g/mL, 10 *μ*g/mL, and 100 *μ*g/mL) were prepared by using ethanol. Ascorbic acid was taken as the standard antioxidant. Briefly, an equal volume of plant extract and DPPH solution (60 *μ*M) were mixed in a test tube. Then, the mixture was left at room temperature in a dark condition for 30 min. Finally, the absorbance was measured at 517 nm using a UV-VIS spectrophotometer.

The percentage of radical scavenging activity was calculated from the formula:

(2) $$\begin{array}{c} \text{DPPH}\text{scavenging}\text{activity}\left(\%\right)=\frac{\left(A_{0}-A_{1}\right)}{A_{0}}\times 100, \end{array}$$

where *A*_0_ is the absorbance of the control and *A*_1_ is the absorbance of the sample.

The antioxidant activity of each plant sample was expressed in terms of IC_50_, which was calculated from the graph obtained by plotting the inhibition percentage against concentration.

### 2.7. Statistical Analysis

All experiments were conducted in triplicate. The results are expressed as the mean ± standarddeviation (SD). The IC_50_ value was calculated using the linear regression equation. The correlations were calculated using Pearson's correlation coefficient (*R*) and coefficient of determination (*R*^2^). A statistical significance of *P* < 0.05 was considered significant. All statistical analyses were conducted using Microsoft Office Excel 2007 and Statistical Package for Social Sciences (SPSS) 16.0 for Windows.

## 3. Results and Discussion

### 3.1. Extraction and Extraction Yield

Extraction yield and efficiency depend on various parameters such as solvent, method, time, temperature, and sample to solvent ratio [25, 26]. The selection of suitable extraction techniques and solvent is a vital step in the screening of bioactive compounds and investigation of biological activities of plant samples. Several studies have reported 70% ethanol as the best solvent in the extraction of a phenolic compound rich extract with potent antioxidant activity [26–28]. Therefore, with the consideration of the objective of the present study, 70% ethanol was selected as a solvent in the extraction.

The extraction yield of various plant samples is given in Table 2. Among the different plant samples tested, the extraction yield was found highest in *F. glaberrima* and lowest in *A. erubescens.* The extraction yield of *F. glaberrima* was almost 2.6 times greater than *A. erubescens.* The reasons behind these differences in the extraction yield values are attributed to the fact that there is a variation in bioactive compounds in between plant samples and these bioactive compounds have different solubility profiles in the extraction solvent. The greater the solubility profile of the bioactive compounds in the extraction solvent, the greater will be the extraction yield value and vice versa [29].

### 3.2. Reducing Sugar Content

Sugar plays a pivotal role in plants as both nutrient and central signaling or regulatory molecules that modulate gene expression related to plant growth, development, metabolism, stress response, and disease resistance. Reducing and nonreducing sugar play an important role in the central metabolic pathways and help in the production of secondary metabolites that enhance the medicinal properties of plants [30, 31]. The quantitative estimation of the reducing sugar content was carried out using the DNSA method. This method is simple, inexpensive, sensitive, and able to handle a large number of samples at a time. It is based on the principle of interaction of an alkaline solution of DNSA with reducing sugars (e.g., glucose and fructose), where the 3-nitro group (NOO^−^) of DNSA is reduced to an amino group and the aldehyde group of reducing sugar is oxidized to the carboxylic acid. In this reaction, orange-red colored 3-amino-5-nitrosalicylic acid is formed which has an absorbance maximum at 540 nm. The intensity of the orange-red color is an index of reducing sugar [32, 33].

The RSC was calculated from the calibration curve (*Y* = 0.00058*x* + 0.002, *R*^2^ = 0.995) of standard D-glucose and expressed as mg GE/g dry extract weight. The RSC of the studied plant samples ranged from 6.89 ± 2.44 to 674.13 ± 2.43 mg GE/g dry extract weight (Table 2). Among the seven different plant samples studied, reducing sugar content was found highest in *F. glaberrima* (674.13 ± 2.43 mg GE/g dry extract weight), followed by *M. malabathricum* (455.16 ± 2.43 mg GE/g dry extract weight), and found lowest in *C. thevetia* (6.89 ± 2.44 mg GE/g dry extract weight), followed by *A. bidentata* (48.27 ± 4.87 mg GE/g dry extract weight).

The RSC in the present studied plant samples has not been reported till date. The RSC in plant samples may vary depending upon genotype, age of the plant, soil quality, geographical location, climatic conditions, cultivation method, and abiotic stress [34, 35]. Several studies have presented the application of quantitative estimation of reducing sugar in plants. The determination of RSC in the plant helps to quantify the degree of acrylamide formation in plant-derived food cooked with high heat, which subsequently helps in determining the quality of food and improving the health of an individual [35, 36]. Similarly, RSC in grapes is an essential parameter for determining the alcoholic level of the grape wine and checking the glucide level during the fermentation process [37].

### 3.3. Total Phenolic Content

The quantitative determination of the total phenolic content was carried out using the Folin–Ciocalteu reagent. The TPC was calculated from the calibration curve (*Y* = 0.012*x*-0.045, *R*^2^ = 0.986) of standard gallic acid and expressed as mg GAE/g dry extract weight. The TPC of the studied plant samples ranged from 14.87 ± 0.41 to 281.71 ± 1.47 mg GAE/g dry extract weight (Table 2). Among the seven studied plant samples, TPC was found highest in *F. glaberrima* (281.71 ± 1.47 mg GAE/g dry extract weight), followed by *M. malabathricum* (222.08 ± 0.11 mg GAE/g dry extract weight), and found lowest in *A. erubescens* (14.87 ± 0.41 mg GAE/g dry extract weight), followed by *A. bidentata* (17.53 ± 0.11 mg GAE/g dry extract weight).

In our previous study [38], we reported the presence of TPC of 68.34 ± 0.38 and 51.03 ± 0.27 mg GAE/g dry extract weight in the ethanolic and aqueous extract of stems of *L. neesiana*, respectively. Gan et al. [39] have reported the presence of 1.34 ± 0.06 mg GAE/g dry extract weight in 80% methanolic extract of *A. bidentata.* Sharma and Kumar [40] revealed the presence of 210 ± 2.99 mg GAE/g of plant extract in methanolic extract of *M. malabathricum* leaves. The differences in the results of TPC between the present and previous studies may be due to the variation in the extraction solvent, part of the plant used, method of analysis, environmental stress, and climatic and geographical conditions [41].

### 3.4. Correlation Analysis between TPC and RSC

**Figure 1:**
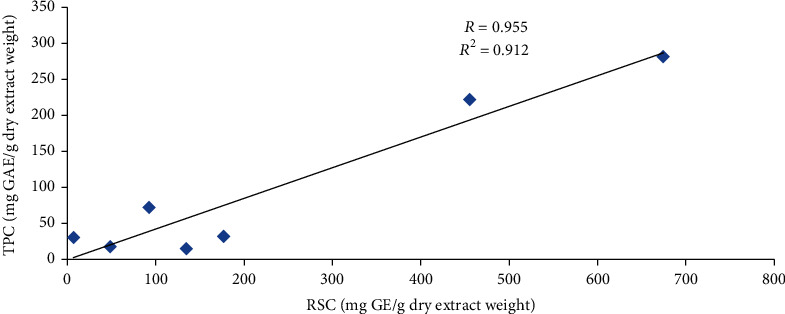
Graphical representation of the correlation between TPC and RSC.

The correlation between TPC and RSC is shown in Figure 1. Pearson's correlation coefficient (*R*) and coefficient of determination (*R*^2^) were found to be 0.955 and 0.912, respectively. The statistical analysis showed a positive and significant (*P* < 0.05) correlation between TPC and RSC, which supports the role of glucose in the synthesis of various phenolic compounds. The two basic pathways for the synthesis of plant phenolic compounds are the shikimic acid pathway and the acetate-malonate (polyketide) pathway. The shikimic acid pathway requires substrates such as erythrose-4-phosphate from the pentose phosphate pathway and phosphoenolpyruvate from glycolysis. These pathways, which are involved in the synthesis of substrates, utilize glucose as a first pivotal molecule [42]. Several studies [43, 44] have also cited the positive correlation between TPC and RSC.

### 3.5. Antioxidant Activity

**Figure 2:**
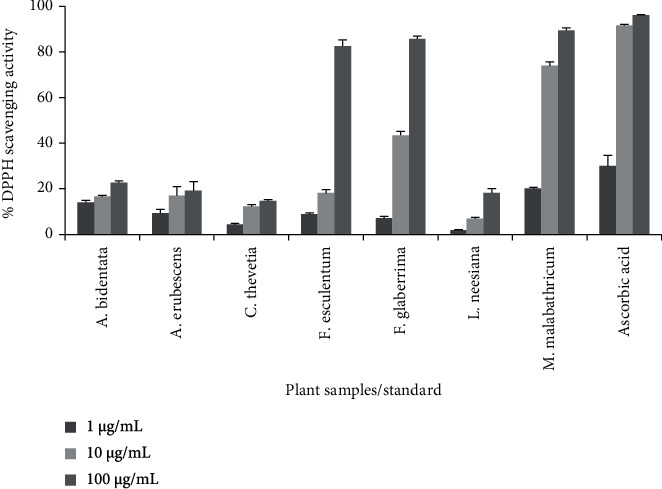
Percentage DPPH radical scavenging activity of selected plant samples and ascorbic acid. The results are expressed as a mean of three replicate determinations(*n* = 3) ± standarddeviation at three different concentrations (1 *μ*g/mL, 10 *μ*g/mL, and 100 *μ*g/mL). Columns represent averages, and error bars represent standard deviations.

DPPH radical scavenging assay is a rapid, inexpensive, and one of the most frequently used methods for evaluating the antioxidant potential of plant extracts. This assay is based on the principle of reduction of purple-colored DPPH solution to the yellow-colored diphenylpicryl hydrazine product in the presence of antioxidants [45]. The DPPH radical scavenging potential of different plant samples is presented in Figure 2.

In the present study, all plant samples showed concentration-dependent DPPH radical scavenging activity. Among the studied plant samples, the highest antioxidant potential was exhibited by *M. malabathricum* (IC_50_value = 6.27 *μ*g/mL), followed by *F. glaberrima*, and *F. esculentum* with IC_50_ values of 11.7 and 55.98 *μ*g/mL, respectively. The lowest antioxidant activity was exhibited by *C. thevetia* (IC_50_value = 479.77 *μ*g/mL), followed by *A. erubescens*, *A. bidentata*, and *L. neesiana* with IC_50_ values of 376.95, 314.56, and 297.89 *μ*g/mL, respectively. The antioxidant potential of standard ascorbic acid was found to be 4.73 *μ*g/mL.

Although a number of studies [38, 40, 46–49] have been carried out demonstrating the antioxidant potential of various parts of the studied plant using different extraction solvents, the study on the antioxidant potential of 70% ethanolic extract of a selected part of the studied plant samples from Nepal has not been performed yet. In our previous study [38], we reported the antioxidant potential of ethanolic and aqueous extracts of *L. neesiana* stems with IC_50_ values of 175.32 and 187.91 *μ*g/mL, respectively. Similarly, Adhikari-Devkota et al. [46] reported the antioxidant activity of 60% ethanolic extract of leaves and twigs of *L. neesiana* with an IC_50_ value of 29.5 ± 1.04 *μ*g/mL. Sharma and Kumar [40] revealed the antioxidant potential of the methanolic extract of *M. malabathricum* with an IC_50_ value of 21.86 ± 0.625 *μ*g/mL. The variation in the results of antioxidant activity of the plant samples between the present and previous studies may be due to the differences in extraction solvent/technique, parts of the plant used, genotype, elevation, soil characteristics, and climatic conditions [50, 51]. To our knowledge, there was no prior scientific report exploring the antioxidant potential, RSC, and TPC of *F. glaberrima.* The present study provides preliminary data on *F. glaberrima* by demonstrating a significant amount of RSC and TPC together with the potent antioxidant capacity. Further studies can be conducted on *F. glaberrima* to explore the biological activities and isolate the chemical constituents.

### 3.6. Correlation Analysis between TPC and Antioxidant Activity

**Figure 3:**
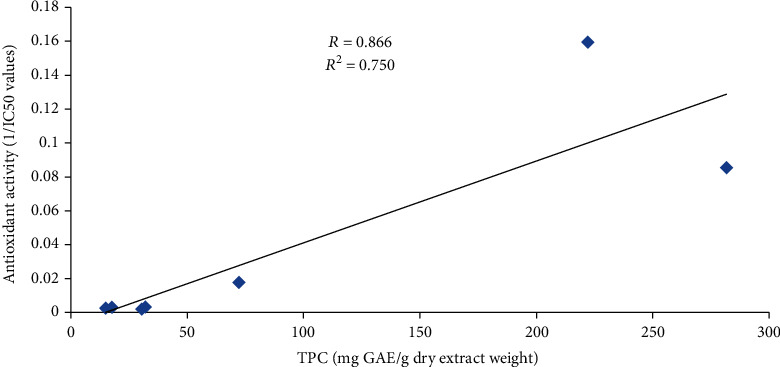
Graphical representation of the correlation between total phenolic content and antioxidant activity.

The correlation of total phenolic content with DPPH radical scavenging activity is shown in Figure 3. Pearson's correlation coefficient (*R*) and coefficient of determination (*R*^2^) were found to be 0.866 and 0.750, respectively. The statistical analysis showed a positive and significant (*P* < 0.05) correlation between the total phenolic content and antioxidant activity, which indicates the possible contribution of total phenolic content in the antioxidant activity of plant samples. Many other studies [52, 53] have also reported the positive correlation between the total phenolic content and antioxidant activity.

## 4. Conclusion

The present study demonstrated the reducing sugar, total phenolic content, and antioxidant potential of the seven plants grown in Nepal. A positive and significant correlation was established between (i) reducing sugar content and total phenolic content and (ii) total phenolic content and antioxidant activity. This is the first scientific report on reducing sugar content of the studied plant samples and the first scientific exploration of *F. glaberrima*. Among the studied plant samples, *F. glaberrima* showed the highest reducing sugar and total phenolic content, together with a greater amount of antioxidant capacity. Thus, these plant samples grown in Nepal can be considered beneficial for the promotion of the health of individuals and be considered a boon to the pharmaceutical industry.

## Figures and Tables

**Table 1:** Scientific names, vernacular names, voucher specimen numbers, parts used, and local uses of selected Nepalese plants.

Scientific names	Vernacular names	Voucher specimen numbers	Parts used	Local uses
*Achyranthes bidentata* Blume	Datiwan	PUCD-2017-14	Stems	Roots juice is used in sore throat and toothache; seeds are useful in piles [14]. Stem is used as a toothbrush and also for the treatment of periodontitis [15].
*Arisaema erubescens* (Wall.) Schott	Sarpako makai	PUCD-2017-15	Tubers	Tubers are used as antihelmintic [16].
*Cascabela thevetia* (L.) Lippold	Karbir	PUCD-2017-17	Leaves	Seeds oil is applied externally to treat skin infection [17].
*Fagopyrum esculentum* Moench	Phaapar	PUCD-2017-24	Grains	Powdered grains are used to treat diarrhoea [18].
*Ficus glaberrima* Blume	Pakhuri	PUCD-2017-25	Bark	Leaves are used as nutritious fodders for cattle [19].
*Lindera neesiana* (Wall. ex Nees) Kurz	Siltimur	PUCD-2017-28	Leaves	Leaves and fruits are used in the treatment of fever, stomach disorder, and skin infection [20].
*Melastoma malabathricum* L.	Angeri	PUCD-2017-29	Bark	Bark paste is used to treat wounds and skin diseases [21].

**Table 2:** Yield percentage, reducing sugar content (RSC), and total phenolic content (TPC) of selected Nepalese plants.

Plant samples	Yield %	RSC (mg GE/g dry extract weight)	TPC (mg GAE/g dry extract weight)
*A. bidentata*	5.7	48.27 ± 4.87	17.53 ± 0.11
*A. erubescens*	3.4	134.47 ± 2.44	14.87 ± 0.41
*C. thevetia*	6.0	6.89 ± 2.44	30.37 ± 2.17
*F. esculentum*	4.25	92.24 ± 6.09	72.12 ± 0.05
*F. glaberrima*	8.95	674.13 ± 2.43	281.71 ± 1.47
*L. neesiana*	5.5	176.72 ± 1.22	31.91 ± 1.18
*M. malabathricum*	8.9	455.16 ± 2.43	222.08 ± 0.11

Each value for RSC and TPC is presented as the mean ± SD (*n* = 3).

## Data Availability

All data generated or analyzed during the present study are included in this article.
